# The Ribosomal Protein RpL22 Interacts In Vitro with 5′-UTR Sequences Found in Some *Drosophila melanogaster* Transposons

**DOI:** 10.3390/genes13020305

**Published:** 2022-02-05

**Authors:** Crescenzio Francesco Minervini, Maria Francesca Berloco, René Massimiliano Marsano, Luigi Viggiano

**Affiliations:** 1 Department of Emergency and Organ Transplantation (D.E.T.O.), Hematology and Stem Cell Transplantation Unit, University of Bari “Aldo Moro”, 70124 Bari, Italy; crescenziofrancesco.minervini@uniba.it; 2 Department of Biology, Università degli Studi di Bari “Aldo Moro”, 70125 Bari, Italy; mariafrancesca.berloco@uniba.it; 3 Department of Genetics Anthropology Evolution, University of Parma, Parco Area delle Scienze 11/A, 43124 Parma, Italy

**Keywords:** ribosomal protein, Rpl22, *Drosophila*, DNA-protein interaction, transposable elements, histone 1-like

## Abstract

Mobility of eukaryotic transposable elements (TEs) are finely regulated to avoid an excessive mutational load caused by their movement. The transposition of retrotransposons is usually regulated through the interaction of host- and TE-encoded proteins, with non-coding regions (LTR and 5′-UTR) of the transposon. Examples of new potent cis-acting sequences, identified and characterized in the non-coding regions of retrotransposons, include the insulator of *gypsy* and Idefix, and the enhancer of *ZAM* of *Drosophila melanogaster*. Recently we have shown that in the 5′-UTR of the LTR-retrotransposon *ZAM* there is a sequence structured in tandem-repeat capable of operating as an insulator both in *Drosophila* (S2R^+^) and human cells (HEK293). Here, we test the hypothesis that tandem repeated 5′-UTR of a different LTR-retrotransposon could accommodate similar regulatory elements. The comparison of the 5′-UTR of some LTR-transposons allowed us to identify a shared motif of 13 bp, called Transposable Element Redundant Motif (TERM). Surprisingly, we demonstrated, by Yeast One-Hybrid assay, that TERM interacts with the *D*. *melanogaster* ribosomal protein RpL22. The *Drosophila* RpL22 has additional Ala-, Lys- and Pro-rich sequences at the amino terminus, which resembles the carboxy-terminal portion of histone H1 and histone H5. For this reason, it has been hypothesized that RpL22 might have two functions, namely the role in organizing the ribosome, and a potential regulatory role involving DNA-binding similar to histone H1, which represses transcription in *Drosophila*. In this paper, we show, by two independent sets of experiments, that DmRpL22 is able to directly and specifically bind DNA of *Drosophila melanogaster*.

## 1. Introduction

Transposable elements (TE) are DNA sequences which are able to move throughout the host genome. These elements were first identified more than 60 years ago by the geneticist Barbara McClintock [1].

TEs constitute a large fraction of the eukaryotic genome (i.e., up to 45% of the human genome and at least 50% of the maize genome [2,3]). The activity of these elements has been linked to more than 75 human diseases including hemophilia A, breast cancer, colorectal cancer, amyotrophic lateral sclerosis, and frontotemporal lobar degeneration [4,5,6,7,8]. In addition, TEs contribute to both neurodevelopment and neurological diseases and disorders [9,10]. Thus, it is important to understand how TEs transpose and how their mobilization is regulated in eukaryotic organisms. While most TEs in the human genome are completely inactive, the thirty percent of the elements in the *Drosophila melanogaster* genome are intact and active [11,12]. As such, *D. melanogaster* has always been considered a model organism for the study of eukaryotic TEs.

TEs are divided into two major classes based on their mechanism of transposition: DNA transposons and retrotransposons. 

The elements of class I, also known as retrotransposons, are mobilized through a “copy and paste” mechanism according to which an intermediate of RNA is reverse transcribed into a cDNA copy and it is integrated elsewhere in the genome [13].

Retrotransposons include Long Terminal Repeat (LTR) retrotransposons, non-LTR retrotransposons (LINEs and LINE-like elements), and short interspersed nuclear elements (SINEs) [14]. 

The genome may be viewed as an ecosystem inhabited by diverse communities of TEs, which seek to propagate and multiply through sophisticated interactions with each other and with other components of the cell [15].

The transposition of class I TEs and its control take place thanks to the interactions between specific non-coding regions of TEs, tRNAs, self-encoded, and host-encoded molecules, including tRNAs and proteins [16].

These non-coding regions are able to control the transcription of the ORFs present in the transposon, and this determines the regulation of their life cycle itself.

Along with these regulatory elements, mostly located in the LTRs, new classes of functional elements have been identified and characterized, the most important of which is called “insulator”. The characterization of TE-related regulatory sequences can also boost the development of new biotechnological tools [17,18].

One of the first TEs where a potent regulatory element has been characterized was *gypsy*, which harbors an insulator in their 5′-UTR. In a previous article, we have shown that also the 5′-UTR of the LTR-retrotransposon *ZAM* acts as an insulator both in *Drosophila* (S2-R^+^) and human cells (HEK293) [19].

Notably, *ZAM*’s insulator has the same tandemly repeated structure and the same localization (5′-UTR) like *gypsy*’s insulator. 

These observations led us to formulate the hypothesis that the tandem repeat regions present in the 5′-UTR of some other retrotransposons (RTs) could accommodate similar regulatory elements. In a previous paper, we grouped the *D. melanogaster* LTR-retrotransposons into three distinct subsets, based on the presence and the complexity of the repeats in the 5′-UTR [20]. Among the retrotransposons with complex repeats in the 5′-UTR, *Tirant* [21,22], *accord* [12], and *ZAM* [23] were selected due to the greater linguistic complexity and lower AT/GC ratio in their tandem-repeat sequences. In the tandemly repeated region of *ZAM*’s 5′-UTR, we had previously identified for the first time the DNA binding site of the HP1 protein [20]. The binding of HP1 to the 5′-UTR of *ZAM* could have a repressive role, inhibiting the retrotransposition of *ZAM*, possibly by recruiting chromodomain-containing proteins, such as protein of the Polycomb group, and thus burying the TE in a heterochromatic domain. The ability to bind the 5′-UTR of a retrotransposon could then represent a generalized defense mechanism of the genome to keep certain species of retrotransposons under control. With the aim to test this hypothesis, we have identified in the 5′-UTR of *ZAM*, *accord*, and *Tirant* a shared motif of 13 bp that we have called TERM (Transposable Element Redundant Motif).

“In vivo” and “in vitro” experiments demonstrated that TERM specifically interacts with the RpL22 protein. We demonstrate here that the peculiar H1/H5-like N-terminal domain of RpL22 of *D. melanogaster* [24,25] is responsible for binding to the TERM motif. We propose that the nuclear localization of RpL22, demonstrated by immunofluorescence experiments, could be supportive of a possible role of Rpl22 as a candidate for the role of controller of the activity of a restricted group of retrotransposons carrying TERM.

## 2. Materials and Methods

### 2.1. In Silico Analysis

Multiple alignments were performed using Multalin [26]. The TERM motif has been detected by using DNA pattern discovery programs which use either enumerative algorithms to examine all oligomers of a given length, reporting those that occur more often than expected as output, or alignment methods to identify unknown signals by local multiple alignment of submitted sequences. We used both approaches to analyze 5′-UTRs of the RTs *accord*, *Tirant*, and *ZAM* using the programs oligo-analysis [27], and MEME [28]. The analyses resulted in similar patterns that can be suitably described by the TERM position weight matrix.

Pattern search. DNA pattern search programs are based on a positional weight matrix (PWM) description of the pattern to be searched. The weight score associated with each examined DNA segment represents a measure of its similarity to the collection of sequences that constitute the PWM—the more a given DNA segment matches the PWM, the higher its weight score. We used the Matrix-scan program [29] to scan the comparable random sequences, generated using *D. melanogaster* as background model with TERM PWM. Analyses were performed with a weight score threshold of 5.29, established as the lower value that is associated with a conserved TERM element in the 5′-UTR of *ZAM*, *accord*, and *Tirant*. 

### 2.2. Plasmid Construction and Sequencing

The yeast integration and reporter vector used to produce the one-hybrid reporter plasmid was pHISi-1 (Clontech, Palo Alto, CA, USA). The reporter plasmid (pTERM3-HISi-1) was constructed by cloning the couple of annealed anticomplementary primers, containing TERM3, into EcoRI/XbaI sites of pHISi-1. 

TERM3-F→5′-aattcATCAAtcgctgaTATCAAtcgctgaTATCAAtcgctgaTg-3′

TERM3-R→3′-gTAGTTAGCGACTATAGTTAGCGACTATAGTTAGCGACTA agatc-5′

Plasmid’s map and corresponding nucleotide sequence are available from the authors.

### 2.3. Yeast One-Hybrid Assay

One-hybrid selection was performed according to the manufacturer’s protocol (Clontech’s MATCHMAKER One Hybrid System) as already depicted in Minervini et al. 2007 [20].

### 2.4. Expression and Purification of RpL22 Protein and RpL22/H5 RpL22/L22 Polypeptides

**Figure 1:**
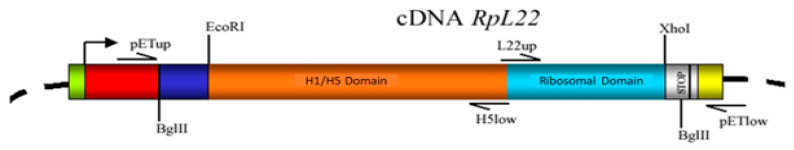
Full-length RpL22 cDNA clone. Graphical representation of the RpL22 cDNA clone which was used to construct vectors expressing RpL22 and its sub-domains (ribosomal and histone-like). Arrows indicate the name and position of the PCR oligo-primers.

The full-length cDNA of RpL22 gene was amplified by High-Fidelity PCR using primer pair pETup/pETlow from one of the Yeast One-Hybrid assay positive clones (Figure 1).

The purified PCR product was cloned into the pET-200 expression vector (Invitrogen, USA) to obtain the pET-200/RpL22 plasmid. This plasmid was transformed into the *E. coli* BL21 Star™ expression host. RpL22 gene was expressed in BL21 Star ™ following the manufacturer’s instructions (Invitrogen, Waltham, MA, USA). His6-Rpl22 protein was purified from harvested cells using the Ni-NTA Fast Start kit (Qiagen, Hilden, Germany) under native conditions following the manufacturer’s instructions. The molecular mass of the protein was determined by SDS-PAGE (12% (w/v) after staining with Coomassie brilliant blue R-250 The concentrations of the purified protein were determined by the Bradford method [30].

The Histone-like domain and the Ribosomal domain of the RpL22 gene were amplified by high-fidelity PCR using, respectively, the primer pair pETup/H5low and pETlow/L22up and cloned the PCR products into pET200 vector to obtain the plasmid pET-200/RpL22_H5, and pET-200/RpL22_L22. Next, the pET plasmids were transformed into *E. coli* BL21 Star™. To obtain the purified RpL22/H5 and RpL22/L22 polypeptides we followed the same procedures described above.

pETup 5′-CACCATGGCTTACCCATA-3′

pETlow 5′-ATAAAAGAAGGCAAAACGATG-3′

H5low 5′-CTAACGCAGCACGTTCTTCTT-3′

L22up 5′-CACCAAGGTGGTCAAGAAGAA-3′

### 2.5. DNA-Binding Assays

Gel mobility shift assays were performed essentially as previously described [31]. Unspecific λ-DNA was sonicated to obtain DNA of average fragments size comparable to that of TERM3.

### 2.6. Production of Antibody Anti RpL22/H5 

Detection of RpL22/H5 polypeptides in Sodium Dodecyl Sulfate-Polyacrylamide Gels was performed as previously described [32].

Briefly, the preparation of RpL22/H5 polypeptide from BL21 lysates has been performed by using sodium dodecyl sulfate-polyacrylamide gel electrophoresis (SDS-PAGE). After electrophoresis, the band of RpL22/H5 polypeptide has been located in the gel by light staining of the gel with 0.05% Coomassie Brilliant Blue R-250 prepared in water. After 10 min of staining the gel has been washed with numerous changes of water over the next few hours. Once the appropriate band was visible, it has been excised with a scalpel.

Preparation of RpL22/H5 polypeptides from Sodium Dodecyl Sulfate-Polyacrylamide Gels for Immunization was performed as previously described [33].

Briefly, after removing the RpL22/H5 band from a polyacrylamide gel, it needs to be fragmented into small pieces before being injected into animals, making it more easily phagocytized and presented to cells of the immune system. We removed the plungers from the barrels of two 5-mL syringes and place the gel fragment into one of the barrels. Afterwards we replaced the plunger and place the syringe outlet in the barrel of the second syringe. Using firm and rapid pressure on the plunger, we pushed the gel into the second syringe. We repeated the process five times, passing the gel fragments back and forth between the two syringes. Finally, we placed 21-gauge needles onto the outlet of the syringes and repeat the process a couple of times. After preparing the antigen (RpL22/H5 polypeptide) we sent it to Invitrogen Custom Polyclonal Antibody Service to obtain the production and purification of the Anti-RpL22/H5 antibody from rabbit.

### 2.7. Immunofluorescence and Immunocytochemistry

Immunofluorescence (IF) and immunocytochemistry (ICC) were performed as follows. Cells were fixed with 4% paraformaldehyde (PFA) for 10 min at room temperature and permeabilized with 0.2% Triton X-100 before immunostaining. The cells were washed in PBS and blocked for 1 h in blocking buffer (10% goat serum in PBS). Samples were incubated with Anti-Fibrillarin antibody (G-8) (sc-374022_Santa Cruz Biotechnology), Anti-Ribosomal ProteinL28 (A-16) (sc-14151_Santa Cruz Biotechnology), Anti-Histone H1 (AE-4) (sc-8030_Santa Cruz Biotechnology), and our Anti RpL22/H5 for 1 h at r.t., washed three times in PBS and incubated with Alexa Fluor 488 goat anti-rabbit secondary antibody (Life Technologies Carlsbad, CA, USA, 1:200 dilution) and Alexa Fluor 488 goat anti-mouse secondary antibody (Life Technologies, Carlsbad, CA, USA 1:200 dilution) for 1 h at r.t. for detection. Counterstaining was done with DAPI. Images were acquired using a Leica IL MD LED inverted fluorescence microscope. To ensure the validity and specificity of the anti RpL22/H5 antibody, we conducted IF and ICC experiments using the pre-immune serum under the same experimental conditions described above without obtaining any signal on the tested cells or tissues.

## 3. Results

### 3.1. Search for Shared Motifs

In a previous work, we published the comparative analysis of the 5′-UTR of the known LTR-retrotransposons of *D. melanogaster* [19]. This analysis revealed that 19 out of 49 5′-UTRs tested (39%) have a tandemly repeated organization and that it was possible to cluster them based on their linguistic complexity and A/T content. It was possible to cluster *accord*, *Tirant*, and *ZAM* into the same group, that share very complex and extended tandemly repeated regions in the 5′-UTRs.

Using the software oligo-analysis, available in the RSATools package [27] (available online: http://rsat.ulb.ac.be/rsat; accessed on 25 April 2018) we performed an analysis to find shared motifs among all tandem repeats identified in the 5′-UTR of the retrotransposons analyzed.

**Figure 2:**
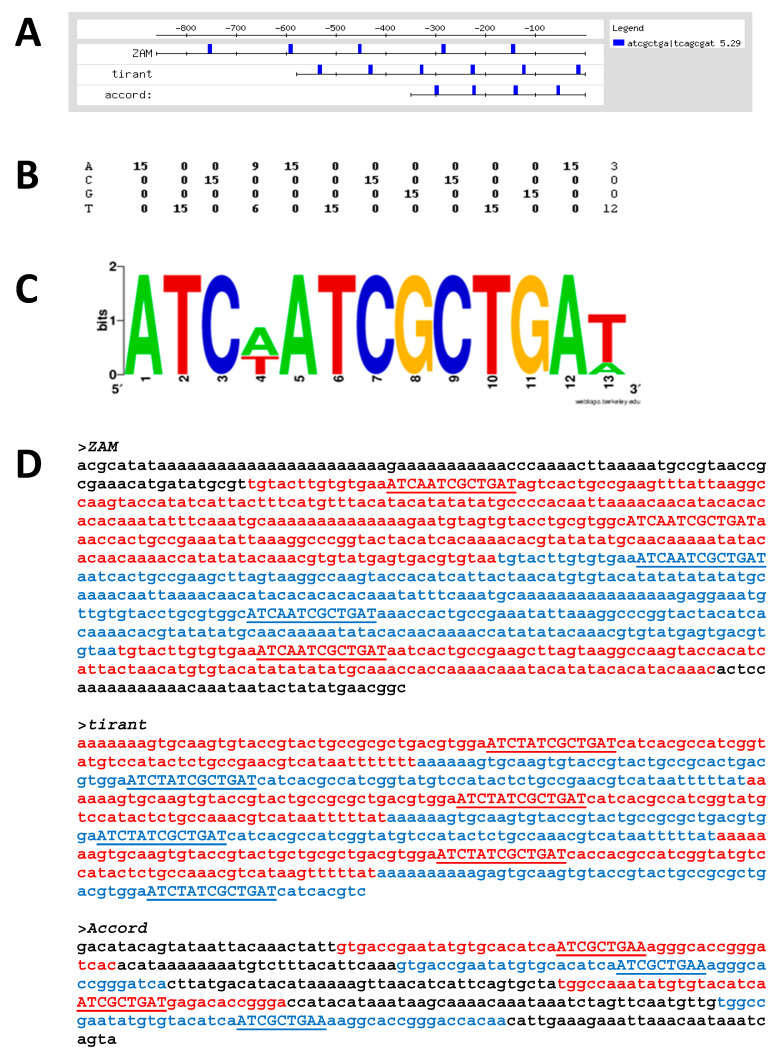
Bioinformatics analysis of the 5′-UTRs of *ZAM*, *Tirant*, and Accord. (**A**) Feature map of over-represented TERM in the 5-UTRs of the indicated RTs. The scale bar provides coordinates relative to the first ORF (GAG) start of the retrotransposons. Note the regularity of the TERM motif in the 5′-UTRs; (**B**) display of the logos of the TERM motif. The graphic representation was created using WebLogo. Sequence logos are a graphical representation of an alignment of multiple nucleic acid sequences (PWM) developed by Tom Schneider and Mike Stephens [34]. Each logo is made up of stacks of symbols, one stack for each position in the sequence. The overall height of the stack indicates the conservation of the sequence at that location, while the height of the symbols within the stack indicates the relative frequency of each nucleic acid at that location; (**C**) positional weight Matrix of TERM motif; (**D**) sequence of the tandem repeats present in the 5′-UTR of the RTEs under examination. The single tandem repeats are in blue and red, while the TERM motifs are in uppercase underscored.

The output of oligo-analysis describes the consensus sequence of the motifs, their position, and its score value representing the statistical significance of the motif and a graphic representation (Figure 2A).

A 13 bp-long motif shared by the LTR-retrotransposons *ZAM*, *accord*, and *Tirant* shows a very high score value. The consensus sequence of the motif is the following: ATCCATCGCTGAT.

The analysis was repeated using an alternative tool (MEME 26), available in the MEME Suite (available at: http://meme-suite.org/; last accessed 25 April 2018) which gave the same. This motif has been called “TERM” (Transposable Element Redundant Motif).

To exclude that TERM was a pattern emerged by chance, we repeated the same analyzes on a group of comparable random sequences, generated using *D. melanogaster* as background model. The sequence generator used is available in RSATools. The TERM matrix was used to scan the 5′-UTR-comparable random sequences using RSATools Matrix-scan program [29]. This analysis did not produce any statistically significant results. Furthermore, we have scanned the 5′-UTRs of all retrotransposons and we found TERM just in *ZAM*, *accord* e *Tirant*. This data acquires further importance considering that in *D*. *melanogaster* there are several hundred copies of the TERM motif, as highlighted by genome to matrix-scan analysis of the genome. So, while it is a relatively common motif, it is only present in the 5′-UTRs of *ZAM*, *accord*, and *Tirant*.

### 3.2. Identification of Proteins Able to Interact with TERM

One way to determine if a short DNA sequence may have a function is to identify protein(s) interacting with it.

For this purpose, we performed a Yeast One-Hybrid assay using the TERM motif as bait (see Material and Methods). 

To carry out the One-Hybrid assay with TERM, we designed a pair of complementary oligonucleotides in which the TERM element was repeated three times (TERM^3^). The double stranded fragment obtained from the annealing of the two oligonucleotides was cloned into the One-Hybrid vectors.

His^−^ yeast mutant strain (YM427), bearing pTERM^3^-HISi-1, the reporter plasmid carrying three TERM tandem-repeat copies cloned upstream of the HIS3 selectable marker, was transformed with a cDNA recombinant plasmid library obtained from 0–21 h embryos of *D. melanogaster* fused with yeast GAL4 activation domain. 

The screening of approximately 8.3 × 10^5^ yeast transformed cells led to the identification of 51 full-length cDNA clones that reproducibly shown to restore the His+ phenotype in yeast bearing the TERM^3^ reporter plasmid, whereas they failed to transform the yeast cells bearing the parental reporter plasmid lacking TERM^3^ to His+ phenotype.

All the positive clones were then sequenced, and the identity of each insert was obtained by a BLAST search of the *D. melanogaster* predicted genes databases. 

The results showed that 35 independent clones (69% of the positive clones) correspond to the gene CG7434 that encodes the Ribosomal protein L22 (RpL22) (see Table 1).

### 3.3. Analysis of RpL22/TERM Interaction

RpL22 encodes for a ribosomal protein of the major subunit of the ribosome in *D. melanogaster*. It encodes a 299 aa-long protein consisting of two domains: a ribosomal L22e C-terminal domain (L22e) which accounts for 1/3 of the protein and a N-terminal H1/H5-like domain (H1/H5) which occupies the remaining 2/3 of the protein [24,25].

The latter domain is a highly basic domain and is characteristic of the H1, and H5 linker histones, and is responsible for their DNA-binding proprieties.

There is no evidence in the literature that RpL22 is able to directly interact with DNA. However, the presence of the H1/H5-like domain, and the result of our Yeast One-Hybrid assay suggest that Rpl22 is a DNA-binding protein. 

Gel mobility shift assays were performed to confirm the ability of RpL22 to bind DNA, and especially the direct interaction between RpL22 and TERM. 

TERM^3^ was terminally radiolabeled and used as substrate. The purified recombinant RpL22 protein was incubated with TERM^3^ and the DNA-protein complexes were resolved by native PAGE. 

**Figure 3:**
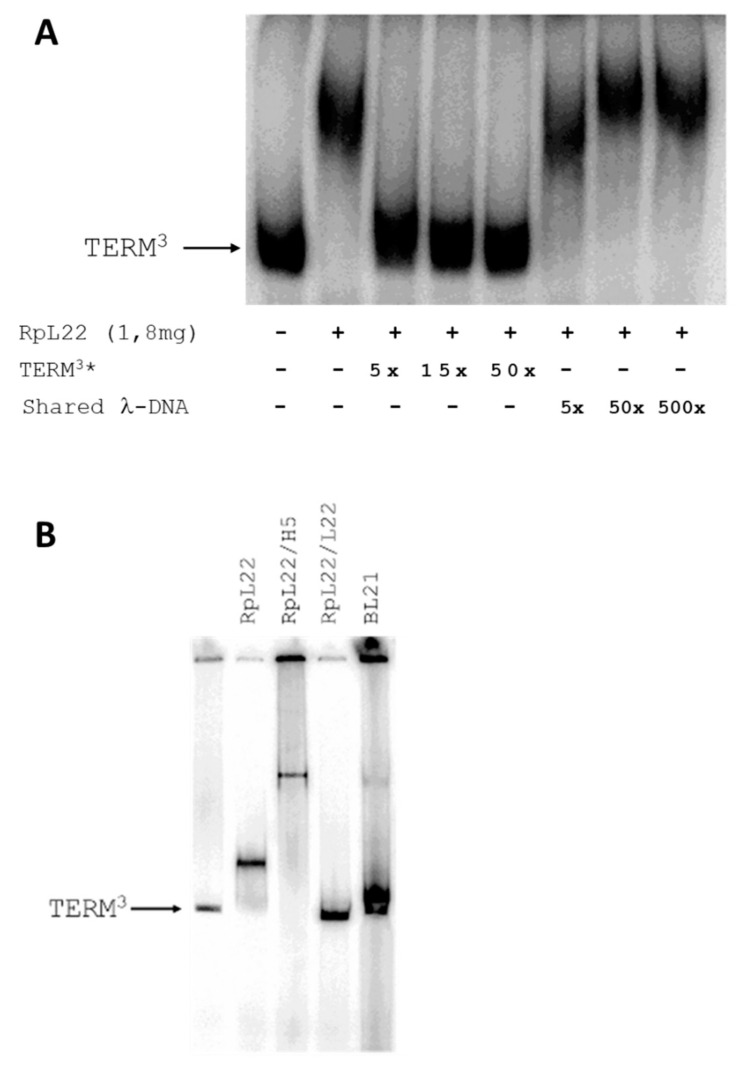
Rpl22 binds the TERM^3^ in vitro. Each lane contains an identical amount of input labeled TERM^3^ DNA (2 ng) incubated with recombinant purified Rpl22 protein. (**A**) TERM3-Rpl22 complex formation has shown in the lane 2, whereas the remaining lanes are committed to specific and not-specific competition experiments: specific competitor (unlabeled TERM^3^*) or a large excess of non-specific competitor (shared λ-DNA) were used as shown in figure; (**B**) identification of which domain of RpL22 is responsible for binding with TERM^3^: we used purified Rpl22 (1.8 µg), RpL22/H5 (1.2 µg), and RpL22/L22 (0.6 µg). We used different amounts of the proteins to maintain the same stoichiometric ratio. The experiment suggests that only the RpL22/H5 polypeptide is able to bind TERM^3^.

The shifted signals indicated that RpL22, when incubated with TERM^3^, was able to produce a slower migrating complex (Figure 3A). 

Several EMSA competition experiments were performed to investigate the specificity of this interaction. Purified RpL22 was incubated with a constant amount of radiolabeled TERM^3^ and in presence of increasing amounts of unlabeled DNA competitors.

The TERM^3^-RpL22 interaction appears to be specific since using up to 500-fold molar excess of unlabeled non-specific competitor (sonicated λ-DNA) did not affect the shift of the TERM^3^-RpL22 complex (Figure 3A). Specific competition experiments performed with an increasing amount of unlabeled TERM^3^ fragments showed that the TERM^3^-RpL22 complex was easily destroyed by adding small quantities (5x) of specific competitor (Figure 3A).

The EMSA results confirmed our assumption that RpL22 may be able to interact specifically with TERM^3^. To understand which domain was responsible for this interaction, we separately cloned the two domains L22e and H1/H5 in the pET expression vector. The two domains were purified and used in further EMSA experiments (Figure 3B). As shown in the figure, only the H1/H5-like domain is able to bind TERM^3^, while the ribosomal domain (C-term domain) does not interact with TERM^3^. This means that the ability of RpL22 to bind TERM3 is conferred by its H1/H5-like domain.

It is interesting to note that the size of the shift, obtained using the entire protein, is lower than that obtained using the H1/H5 domain alone. Apparently, this seems to be inconsistent, as we would expect an opposite behavior, given the reduction in the size of the protein. We believe that the lack of the L22 domain causes a change in the charge density of the polypeptide, which becomes more positive and this slows the run of Rpl22/H5 polypeptide.

### 3.4. RpL22 Sub-Cellular Localization

From what we have shown so far, it emerges that the RpL22 protein has a bivalent nature with an additional non-ribosomal function. It is indeed composed of a Histone-like portion (N-term) and a ribosomal portion (C-term). Consistent with the presence of the histone portion, RpL22 is able to interact with DNA both “in vivo” (Yeast One-Hybrid assay) and “in vitro” (EMSA).

To try to uncover in vivo in *D. melanogaster* the extra-ribosomal role of Rpl22, we analyzed its cellular location.

Mageeney and colleagues showed that RpL22, in the testes, displays a punctate nuclear pattern, probably in the nucleoli [35].

Given the nature of the tissue examined, together with the fact that the expression of the RpL22 gene is not ubiquitous in all testicular cell subtypes, we wanted to verify the protein sub-cellular localization in a more tractable experimental cellular system, such as the *D. melanogaster* S2R^+^ cell line.

**Figure 4:**
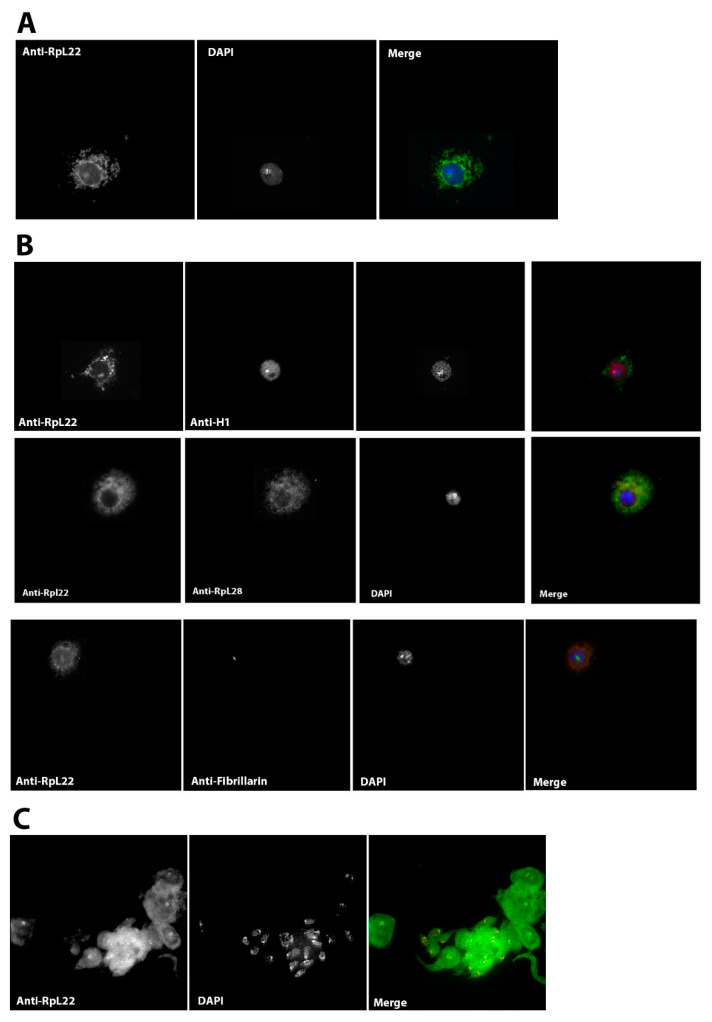
RpL22 localization in *Drosophila* cell line S2R^+^ and in the brain cells. (**A**) Rpl22 localizes both in cytoplasm and nucleolus in S2R^+^ cell. (**B**) To highlight the “ribosomal” behavior of RpL22, co-immunofluorescence experiments were performed both with the anti-H1 antibody and anti-RpL28 antibody, finally, as further confirmation of the nucleolar localization, RpL22 co-localizes with the nucleolar marker of fibrillarin. (**C**) The same localization pattern occurs (cytoplasm and nucleolus) also in neurons.

We produced a polyclonal antibody from the H1/H5-like domain to be used in immunofluorescence experiments on S2R^+^ cells. To allow a more precise sub-cellular localization of the RpL22 protein, we performed both immunofluorescence and co-immunofluorescence experiments. We used anti-RpL22, anti-Rpl28, anti-H1, and anti-fibrillarin antibodies (Figure 4). Immunofluorescence staining experiments using formaldehyde-fixed S2R^+^ cells showed that RpL22 has the expected ribosomal distribution pattern, namely a cytoplasmic and nucleolar localization (Panel A). Notably, the nucleolar localization of RpL22 roughly corresponds to DAPI staining loss in the nucleus. The pattern of RpL22 corresponds to the pattern of other ribosomal proteins (such as RpL28) (Panel B). We also performed co-immunofluorescence staining experiments using anti-RpL22 and anti-H1 antibodies. As expected, the two proteins have very different and almost specular localization, while RpL22 is localized in the cytoplasm and the nucleolus, H1 is positioned only in the nucleus with a staining free region that corresponds to the volume occupied by RpL22 in the nucleolus (Panel B).

To confirm the cellular and subcellular localization of RpL22 also in tissues other than cultured cells and germline tissues, we performed immunofluorescence experiments also on *Drosophila* brain tissue, confirming the same cytoplasmic and nucleolar localization of RpL22 already highlighted in S2R^+^ cells (panel C).

## 4. Discussion

The relationship between retrotransposons and the host genome has a double nature. On the one hand, RTs are a source of genetic variability that has been exploited during the evolution to develop both metabolic and physiological innovations such as the placental syncytins [36], or they are involved in genomic stress response and adaptation, mainly through rewiring of transcriptional networks [16,37,38]. On the other hand, retrotransposons constitute a hazard both to the structural integrity of the genome and its functionality. To balance these two opposite conditions, the eukaryotic genome has evolved mechanisms to control transposition and exploit RT genes and their regulatory regions to develop new gene functions (gain of function). Eukaryotic cells have developed a series of transcriptional repression mechanisms to tame retrotransposons, basically based on small RNAs. *gypsy* is controlled by a *Drosophila* locus called *flamenco*, which maintains the retrovirus in a repressed state. The *flamenco* locus acts as a source of piRNAs in the ovary, while in somatic tissues it acts as a source of endo-siRNAs [39,40,41].

Alternative mechanisms for controlling RTs activity have also been proposed, for example the transposon homing [42]. It has been hypothesized that the interaction between a motif present in the 5′-UTR of *ZAM* and the HP1 protein allows cells to direct the insertion of *ZAM* in a biased manner into the genome. This would force the new transposed copies of *ZAM* to converge in the heterochromatic regions of the genome where they would remain inactive [19].

However, the host-RT co-evolution has sometimes resulted in the development of potent regulatory sequences within the non-coding regions of RTs, due to their physical interaction with host factors. This phenomenon has been used by the host to create additional variability associated with the transcriptional rewiring of gene networks.

The 5′-UTR regions of some RTs are of particular interest from this standpoint. The 5′-UTR of RTs like *gypsy*, *ZAM*, *Tirant*, and *Idefix* have a polymorphic repeated organization. In some cases, the repeated structure has been associated with enhancer (*ZAM*), silencer (*gypsy*), or insulator (*gypsy*, *ZAM* and *Idefix*) functions. These functions are fulfilled through the physical interaction of one or more host proteins with the 5′-UTR of the RT, as reported for *gypsy* [43,44] and *ZAM* [20]. We have therefore tested the hypothesis that RTs harboring a structured 5′-UTR could be bounded by host proteins that could either regulate their transposition or confer them new functions.

In this work, we first identified a shared motif (TERM) in the repeat-containing 5′-UTRs of some RTs using a comparative analysis approach, and subsequently found the RpL22 protein as the main interacting protein of the TER motif, using the Yeast One-Hybrid assay. We have finally confirmed the Rpl22/TERM interaction in vitro and mapped the DNA binding domain to the NH-terminal portion of the protein. Although our IF experiments are not informative of the Rpl22/DNA interaction in vivo, this connection cannot be excluded, as discussed below.

So, what is the biological relevance of our findings? RpL22 is a ribosomal protein mainly localized in the cytoplasm. Nevertheless, other studies have highlighted its role in establishing a state of generalized transcriptional repression [45], as already demonstrated for histone H1 [45]. The results of our experiments show that not only RpL22 can interact directly with DNA, but also that this interaction is sequence-specific (TERM motif). The fact that RpL22 possesses a histone H1/H5-like domain capable of binding DNA leads us to hypothesize that the RpL22 protein could act, through the binding to the TERM motif, as a transcriptional repressor, especially on *ZAM*, *accord*, and *Tirant* where several copies of TERM are clustered (Figure 2A,D).

Although we have found that the Histone-like region is responsible for the binding of RpL22 to TERM both in vivo (Yeast One-Hybrid assay) and in vitro (EMSA), unlike Ni and colleagues [45], we were not able to pinpoint a chromosomal localization of RpL22 by IF and ICC experiments except for the nucleolus region (Figure 4). This may be due to the cell type (S2R^+^), or tissue (*Drosophila* brain) used in our study.

**Figure 5:**
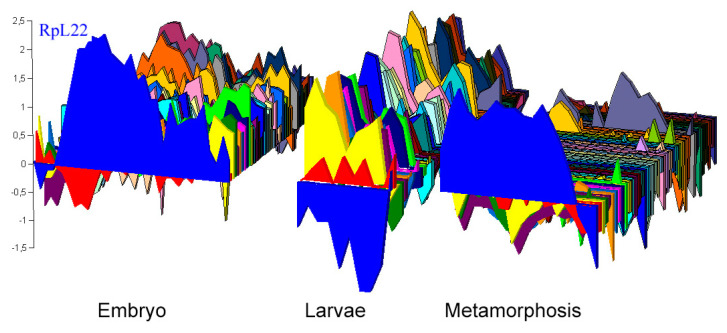
Comparison of the expression profile of RpL22 (in blue) with the other ribosomal proteins during the development of *Drosophila melanogaster*. Microarray data of ribosomal *D. melanogaster* gene expression during development was downloaded from the FLYMINE database [49] (available at: https://www.flymine.org/flymine; last accessed 15 December 2021). These data were used to construct the graph. Y axis: fold change. Reference sample is a pooled mRNA representing all stages of the life cycle as reported in Arbeitman et al. [48].

The behavior of RpL22 may depend on the cell type. Some post-translational modifications of RpL22 (SUMOylation and phosphorylation) are known [46], and they may be able to modify the localization and/or function of RpL22 in a tissue- and/or developmental stage-dependent manner. It could also be hypothesized that, in S2R^+^, neuron, and salivary gland cells [47], the putative chromatin-associated function of Rpl22 could be dispensable, while it could be essential in other tissues not investigated in this study. Imaginal discs are tissues experiencing profound changes in the transcriptional program and Rpl22 is one of the very few ribosomal genes active during metamorphosis (Figure 5) [48]. Therefore, Rpl22 might exert its role in controlling TEs, during the metamorphosis.

In a parallel study [47], we have also demonstrated that the *Doc5* transposon, which is located exquisitely in the heterochromatin of *D. melanogaster*, is also a binding site for Rpl22. Being a LINE-like transposon, *Doc5* has not been included in this study. At least six TERM-like motifs can be found in the *Doc5* sequence (Figure S1), which suggests that Rpl22 exhibits sequence specificity. Moreover, the study by Berloco et al. [47] confirms the connection between Rpl22 and transposable elements.

Additional studies, aimed at the identification of the Rpl22/DNA interaction in vivo are required to support our current hypotheses. However, studies aimed at revealing Rpl22 as a chromatin component require antibody optimization and the development of a transgenic line that expresses efficiently the Rpl22 protein. The only transgenic line allowing the overexpression of Rpl22 available to date [50] does not allow for an efficient testing of our hypothesis. Furthermore, it is possible that the Rpl22 binding to chromosomes could be only highlighted in vivo under particular physiological conditions (such as development, tissue, or stress specific conditions), making it difficult to uncover the role of Rpl22 in chromatin dynamics. 

In conclusion, our results show that the *D. melanogaster* Rpl22 protein specifically interacts in vitro with DNA sequences related to TEs. While our findings open up the possibility for RpL22 to participate in controlling TEs of *D. melanogaster*, such interactions need an experimental validation in vivo using specific approaches such as Chip-seq or similar methods.

## Figures and Tables

**Table 1:** Yeast One-Hybrid assay results.

Clone’s Name	BLAST Results	BLASTX Results	Notes
*L1*	mitochondrial sequence	#	#
*L2*	Grn	GRN	GATA trascription factor
*L3*	CG7434	RpL22	Ribosomal protein
*L4*	CG7434	RpL22	Ribosomal protein
*L5*	Csn6	CSN6	Signalosome
*L6*	CG7434	RpL22	Ribosomal protein
*L7*	Mis	MIS	body pigmentation
*pTERM3lig01*	CG4314	st	eye pigment precursor transport
*pTERM3lig02*	GS1	GS1	glutammina sintetasi
*pTERM3lig03*	CG1883	CG1883	Rps7-like
*pTERM3lig05*	CG7434	RpL22	Ribosomal protein
*pTERM3lig06*	CG7434	RpL22	Ribosomal protein
*pTERM3lig07*	Hrb27C	Hrb27C	RNA binding protein
*pTERM3lig08*	CG7434	RpL22	Ribosomal protein
*pTERM3lig09*	CG7434	RpL22	Ribosomal protein
*pTERM3lig10*	CG7434	RpL22	Ribosomal protein
*pTERM3lig11*	CG9253	CG9253	RNA helicase activity
*pTERM3lig12*	Mod(mdg4)	Mod(mdg4)	FLYWCH domain
*pTERM3lig13*	CG7434	RpL22	Ribosomal protein
*pTERM3lig14*	CG7434	RpL22	Ribosomal protein
*pTERM3lig15*	RpS16	RpS16	Ribosomal protein
*pTERM3lig16*	CG7434	RpL22	Ribosomal protein
*pTERM3lig17*	CG7434	RpL22	Ribosomal protein
*pTERM3lig19*	CG7434	RpL22	Ribosomal protein
*pTERM3lig20*	CG7434	RpL22	Ribosomal protein
*pTERM3lig21*	CG7434	RpL22	Ribosomal protein
*pTERM3lig25*	CG7434	RpL22	Ribosomal protein
*pTERM3lig26*	CG7434	RpL22	Ribosomal protein
*pTERM3lig27*	CG6007	GatA	serine hydrolase activity
*pTERM3lig29*	CG7434	RpL22	Ribosomal protein
*pTERM3lig31*	CG7434	RpL22	Ribosomal protein
*pTERM3lig32*	CG30389	CG30389	actin filament binding activity
*pTERM3lig33*	CG7434	RpL22	Ribosomal protein
*pTERM3lig34*	CG7434	RpL22	Ribosomal protein
*pTERM3lig35*	CG9415	CG9415	trascription factor
*pTERM3lig36*	CG9277	CG9277	beta tubulina
*pTERM3lig38*	CG7434	RpL22	Ribosomal protein
*pTERM3lig39*	CG7434	RpL22	Ribosomal protein
*pTERM3lig41*	CG7434	RpL22	Ribosomal protein
*pTERM3lig43*	CG7434	RpL22	Ribosomal protein
*pTERM3lig44*	CG7434	RpL22	Ribosomal protein
*pTERM3lig45*	CG7434	RpL22	Ribosomal protein
*pTERM3lig46*	CG7434	RpL22	Ribosomal protein
*pTERM3lig47*	CG7434	RpL22	Ribosomal protein
*pTERM3lig48*	CG7434	RpL22	Ribosomal protein
*pTERM3lig49*	CG7434	RpL22	Ribosomal protein
*pTERM3lig50*	CG7434	RpL22	Ribosomal protein
*pTERM3lig51*	CG17326	luna	Zinc finger C2H2-type
*pTERM3lig52*	CG7434	RpL22	Ribosomal protein
*pTERM3lig53*	CG7434	RpL22	Ribosomal protein
*pTERM3lig54*	*CG7434*	RpL22	Ribosomal protein

Fifty-nine percent (35 out of 51) of the positive clones isolated in the One-Hybrid assay correspond to gene CG7434 (in green) encoding the ribosomal protein RpL22. The remaining 16 clones correspond to non-coding mitochondrial sequences (L1), structural or enzymatic proteins (L5, L7, pTERM3lig01, pTERM3lig02, pTERM3lig36, pTERM3lig27), other ribosomal protein (pTERM3lig15), and some transcription factors and/or other DNA binding proteins (L2, pTERM3lig12, pTERM3lig32, pTERM3lig35, pTERM3lig51). It is important to underline that, except for the CG7434 gene, all clones screened with the One-Hybrid assay are represented only once.

## Data Availability

Not applicable.

## Supplementary Materials

The following supporting information can be downloaded at: https://www.mdpi.com/article/10.3390/genes13020305/s1, Figure S1: Doc5 transposon fragment harbors at least 6 TERM-like elements
