# Supplementary material for: The Ribosomal Protein RpL22 Interacts In Vitro with 5′-UTR Sequences Found in Some Drosophila melanogaster Transposons
Source: Genes (Basel). 2022 Feb 5;13(2):305. doi: 10.3390/genes13020305 (PMC8872304; doi:10.3390/genes13020305)
Supplement: Supplementary file 1 [file genes-13-00305-s001.zip › genes-1474838-supplementary.pdf]

A

aaaccaagtaactagccat**caaaccaactca**acaccttaacttactattgtacctgatatacag
cctgcttcaactcaattaagccaaactgcacaaaatgattttgacgaacttaaacaaatgatga
aaca**attaattgccca**aatgacaaatatgatgaacat**tttctcgctttt**attattcaaacttga
caaataaacacct**aacaatcgccat**ctggaatgctaacggt**ctttcacgccat**ttacatgaact
aaaaacattcttaaatgaaaagcaaattgaggtcatgctcatttctatataaacatacctaact
ataatatttcttctacttatcacccggacggtaaggcagtaataattaaaaaaaagcatcaagt
gcatagagctcgatggattcaaaaaggactatatacaggctactactaaatcgacctcagacac
aacgggtccaataaatatatctgcagtttatttttcacctaaatttaataatacaaaagaccaa
tacctcgatttcctaaaatcactgggaaatcggtactttgcaggaggtgactataa**tccaaaca**
cacaacttgggggtccagac

B

```
              1        10   15
              |--------+----|
TERM-like1      AACAATCGCCAT
TERM-like2    CAATTAATTGCCCAA
TERM-like3    GTCTTTCACGCCA
TERM-like4       TTTCTCGCTTTT
TERM-like5     TCCAAACA-CACAA
TERM-like6    ATCAAACCAACTCAA
 Consensus    ...ttaatcgCc...
```

C

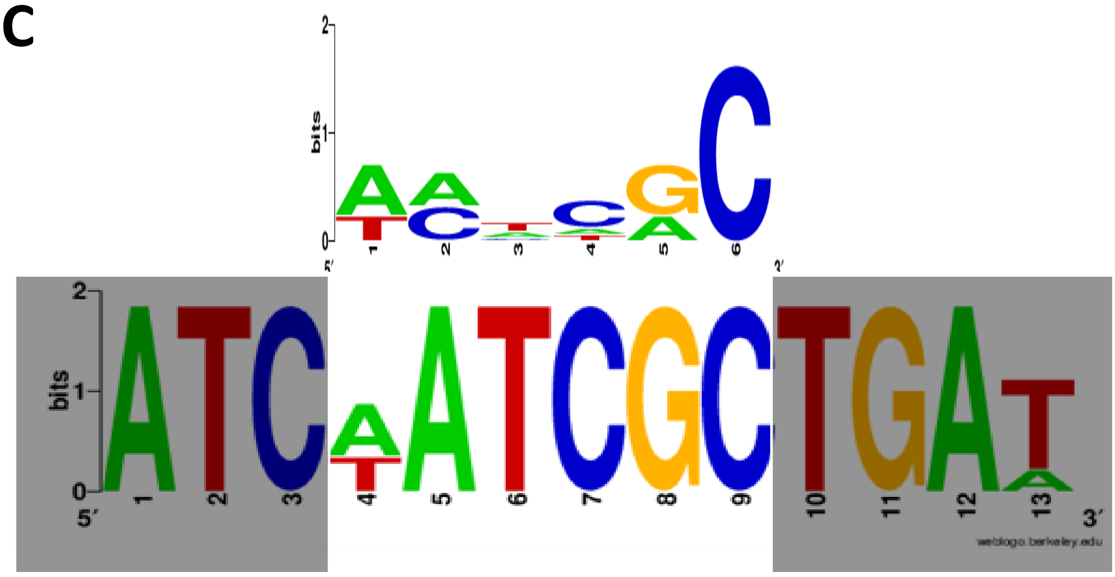

Figure S1. Doc5 transposon fragment harbours at least 6 TERM-like elements
A) Sequence of the Doc5 fragment analyzed in Berloco et al. 2021. The TERM-like motifs are in red underscored fonts; B) The multiple alignment (Multalin) of TERM-like sequences with the consensus sequence; C) Display of the logos of the TERM-like sequences in Doc5 (top) compared to logos of TERM motif (bottom). From the comparison of the Weblogos it emerges that TERM and the TERM-like consensus share the central portion.
